# Advances in the Interpretation of the Electrocardiogram by Artificial Intelligence

**DOI:** 10.3390/diagnostics16142167

**Published:** 2026-07-10

**Authors:** S. Suave Lobodzinski, Ryszard Piotrowicz

**Affiliations:** 1 School of Medicine, University of California, Los Angeles, CA 90095, USA; 2 Department of Electrical Engineering, California State University, Long Beach, CA 90840, USA; 3 National Institute of Cardiology, 04-628 Warsaw, Poland

**Keywords:** artificial intelligence, electrocardiography, deep learning, arrhythmia detection, digital biomarkers, clinical decision support, cardiovascular diagnostics, AI-ECG

## Abstract

The electrocardiogram (ECG) is essential for cardiovascular diagnosis but limited by inter-observer variability, low sensitivity for subclinical disease, and labor-intensive telemonitoring analysis. Artificial intelligence (AI), particularly deep learning, addresses these constraints by extracting high-dimensional patterns that correlate with arrhythmias, structural abnormalities, and systemic conditions. This integrative review synthesizes recent advances in AI-enabled ECG, covering technical foundations—including foundation models and validation strategies—and clinical applications, such as arrhythmia detection, structural heart disease identification, and digital biomarker derivation. We discuss emerging trends like self-supervised learning, multimodal integration, generative models, and explainability techniques. Furthermore, we tackle critical challenges regarding generalizability, algorithmic bias, privacy, and regulatory systems. Finally, we outline research priorities, including curated open datasets, and deployment in resource-constrained settings. With stringent validation, transparent governance, and human-centered design, AI-ECG has the potential to enhance cardiovascular diagnostics and clinical outcomes across a variety of healthcare settings.

## 1. Introduction

The electrocardiogram (ECG) continues to be the cornerstone of cardiovascular diagnosis, a century after its inception. Its ubiquity, cost-effectiveness, portability, and ease of acquisition make it vital for acute chest pain triage, arrhythmia evaluation, and the longitudinal management of structural heart disease. Beyond these conventional roles, the ECG encodes high-dimensional information regarding cardiac structure, conduction dynamics, and autonomic tone that can precede overt clinical disease [1]. Exploiting this complex, rich signal is now central to early detection and exact risk stratification in contemporary cardiology.

Despite its clinical importance, conventional ECG interpretation confronts significant limitations. Visual analysis is fundamentally subjective, prone to inter- and intra-observer variability, and frequently lacks the sensitivity to detect subtle findings, such as minor ST-T changes or early conduction disturbances [2]. Diagnostic accuracy is further modulated by clinician training, fatigue, and clinical context, leading to diagnostic inconsistencies. Moreover, human readers are generally ill-equipped to identify the subtle, high-dimensional waveform patterns that correlate with microstructural remodeling or future adverse events. Consequently, the sensitivity of standard interpretation for subclinical cardiomyopathies, incipient left ventricular (LV) dysfunction, or future atrial fibrillation (AF) remains modest [3,4,5].

Many clinical workflows rely on rule-based automated ECG interpretation followed by clinician overread—a strategy that is efficient yet has inconsistent accuracy.

A 2025 study published in Frontiers in Physiology [3] reported that 39% of automatically interpreted ECGs were misinterpreted, with both false-negative and false-positive errors occurring frequently. The erroneous interpretations involved ischemic features, conduction abnormalities, rhythm disturbances, and chamber hypertrophy. Diagnostic errors in ECG interpretation have significant clinical and economic consequences: missed diagnoses delay appropriate treatment and may allow disease progression, while overdiagnosis subjects patients to unnecessary anxiety, repeat testing, and avoidable costs, while increasing clinician workload and responsibility.

Kim et al., in a 2022 study published in Clin Exp Emerg Med, reported that automatic electrocardiogram interpretation achieved an overall diagnostic accuracy of 73.5% for ST-segment elevation myocardial infarction, with a misinterpretation rate of 26.5% [2].

The emergence of wearables and long-term Holter monitors for ECG telemonitoring has increased diagnostic yield but has also resulted in labor-intensive analysis of the massive volumes of data generated by continuous recording [6,7].

In the following, we will discuss recent advances in artificial intelligence (AI) that represent a paradigm shift in ECG Interpretation. In contrast to traditional methods, AI algorithms are trained on large-scale, annotated datasets to detect patterns that extend well beyond rule-based features such as intervals, amplitudes, or axes [8]. AI ECG has the potential to substantially improve ECG interpretation by reducing the subjectivity and variability inherent in conventional visual analysis, particularly for subtle or early abnormalities that may be overlooked by human readers. By learning complex waveform patterns from large datasets, AI systems can enhance the detection of conditions such as ischemia, conduction disturbances, left ventricular dysfunction, cardiomyopathy, and atrial fibrillation, while reducing false-positive interpretations that lead to unnecessary testing and clinical burden. When incorporated into routine clinical workflows as decision-support tools, AI-assisted ECG analysis may reduce time to diagnosis, improve interpretive consistency across clinicians and practice settings, and enhance diagnostic accuracy in both screening and high-risk populations, with performance approaching that of cardiologists [9].

This integrative review aims to synthesize recent advances in AI-enhanced electrocardiography and to examine their relevance to clinical practice.

We first summarize the technical foundations of contemporary machine learning approaches, including data requirements, model architectures, and validation strategies. We then discuss established and emerging clinical applications, spanning rhythm disturbances, structural heart disease, and systemic digital biomarkers. Finally, we address critical challenges—including generalizability, algorithmic bias, regulatory governance, clinical integration, and future applications—to provide a concise framework for the responsible translation of AI-ECG into routine cardiovascular care [9,10].

## 2. Methods

In the conceptual stage of this integrative review, we formulated the following research question:

What is the current state of the art of AI technologies as applied to electrocardiology to accurately diagnose arrhythmias, structural heart disease, and systemic health assessment and prognosis; what clinical performance do they achieve; what datasets support their development; and what challenges, regulatory constraints, limitations, ethical considerations, and future directions have been reported?

### 2.1. Selection of Resources

A quick search of PubMed using a simple (AI and ECG) syntax resulted in 1744 records published in the last 10 years. Instead of sorting through all of them, we decided to perform multiple focused searches on topics corresponding to the sections or subsections of the review outline (16 total).

The selection process comprised three stages:

Stage 1—Title/abstract/webpage/search. A total of 740 records were retrieved published between 2020 and 2026, from the following databases: PubMed (*n* = 247), Scopus (*n* = 274), Web of Science (*n* = 127), arXiv (*n* = 65), YouTube (*n* = 27), and web (via Google search). After removing 262 duplicates, 478 unique records were screened for relevance to the outline sections.

Stage 2—Full-text assessment: After the initial screening, 251 records were excluded. The remaining 227 articles were subjected to full-text review by the authors. All discrepancies were resolved through discussion.

Reasons for exclusion included:

Absence of relevance to the specific topic (ex. AI and ECG and External and internal validation);

Reviews, press releases, and commentaries;

Animal/in vitro studies;

Lack of quantitative data or analysis;

Non-English articles.

Stage 3—Assessment of YouTube and web page records. After the initial screening, 9 records were excluded as per the eligibility criteria (Section 2.2), and 88 studies were included in the final qualitative synthesis.

### 2.2. Eligibility Criteria

Inclusion criteria:

Original research studies and commercial AI ECG products relevant to the section of the paper;

Publications available in English with full text accessible;

Web pages describing AI ECG commercial products;

YouTube videos discussing AI ECG models, academic AI ECG presentations, and commercial AI ECG products.

Exclusion criteria:

Conference abstracts, letters, editorials, reviews, or protocols without full primary data, or advertising materials;

In vivo, in silico, or phantom studies;

Studies lacking clinical reference standards (including cardiologist adjudication, imaging, catheterization, or follow-up);

Duplicate publications reporting the same cohort without new methodological or outcome data.

### 2.3. Sources of Data

A literature search was performed in PubMed/MEDLINE, Scopus, Web of Science, arXiv, and YouTube databases. The search period spanned from January 2020 to May 2026. The strategy was constructed around Population, Intervention, Comparison, and Outcome elements and tailored to each database. The core syntax was (electrocardiogram OR ECG OR EKG) AND (AI OR AI ECG) AND (section of the paper topic, e.g., Models (OR Architecture). The YouTube syntax was (AI ECG or EKG).

## 3. Technical Foundations of AI in ECG Analysis

**Figure 1:**
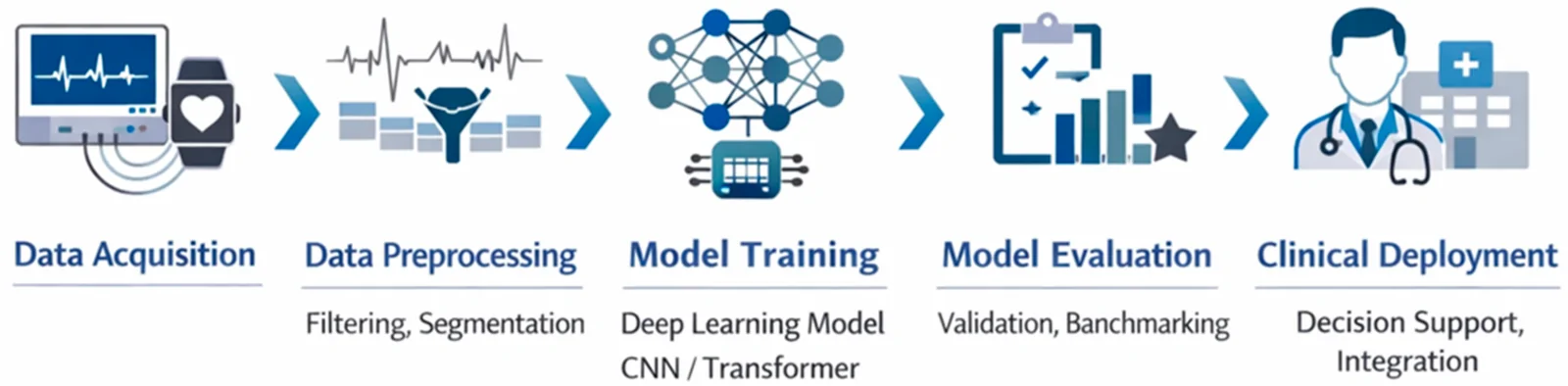
The End-to-End AI-ECG Pipeline is a comprehensive workflow that converts raw electrocardiogram data into clinically actionable diagnostic insights using artificial intelligence. ECG signals are collected from various sources, including digital devices, PDF reports, and equipment from different manufacturers [11]. Digital ECG data is then preprocessed, cleaned, and normalized, noise is filtered out, and data from heterogeneous sources is formatted for machine learning. The model training phase uses deep training architectures (such as convolutional or recurrent neural networks) to learn patterns associated with cardiac pathologies, including myocardial scar, arrhythmias, and long QT syndrome. Finally, the deployment and interpretation stage deliver AI-powered findings integrated with clinical information, frequently incorporating explainable AI features that indicate which ECG segments drove the diagnosis, enabling clinicians to trust and act on the AI’s recommendations. This unified pipeline forms Software as a Medical Device (SaMD), a standalone software that performs ECG processing and interpretation independently, without being part of a hardware ECG machine.

Artificial intelligence methods for electrocardiogram interpretation rest on a set of technical foundations that span data types, model architectures, and end-to-end analytic pipelines as shown in Figure 1. Comprehending these elements is essential for evaluating current systems and designing new ones that are robust, generalizable, and clinically meaningful.

Robust AI-ECG systems depend not only on the choice of architecture but also on a rigorous pipeline for data curation, preprocessing, labeling, and validation. It is a critical step in preparing data stemming from multiple sources for deep learning [12]. Data acquisition typically begins with the extraction of raw digital ECG files and associated metadata from hospital ECG management systems, Holter databases, device interrogations, or wearable platforms, followed by de-identification procedures that remove or encrypt protected health information whilst preserving clinically relevant attributes such as age and sex. Large multi-center ECG projects must harmonize sampling rates, lead configurations, and file formats across vendors to enable joint model training and reduce domain shift, as illustrated by population-scale ECG cohorts that reanalyze recordings with a single standardized algorithm and exclude nonconforming acquisitions [13,14].

### 3.1. Types of ECG Data for AI

Digital ECG signals arise from multiple sources that differ in duration, sampling characteristics, noise profile, and clinical context, all of which shape the design and performance of AI models. Standard 10-s, 12-lead recordings obtained in resting conditions remain the most common input for supervised learning, as they are widely available in hospital archives and are accompanied by diagnostic reports that can be mined at scale for labels. Longer-term Holter and patch recordings provide continuous multi-hour to multi-day monitoring, allowing detection of infrequent arrhythmias and dynamic changes in rate or conduction, but they introduce substantial class imbalance and a higher artifact burden.

Data from implantable devices—such as pacemakers, implantable cardioverter-defibrillators, and implantable loop recorders—typically consist of single- or dual-lead intracardiac electrograms and event-triggered segments, requiring models that can handle sparse, device-specific signals while exploiting precise temporal annotations of arrhythmic episodes. Wearable devices, including smartwatches, fitness bands, and consumer ECG patches, contribute vast volumes of single-lead or reduced-lead signals sampled at varying frequencies and often acquired in nonclinical environments. These data streams broaden population coverage and capture physiology during daily activities, but they introduce challenges related to motion artifacts, variable electrode placement, and uncertain ground truth [15,16,17,18,19,20,21].

Recent foundation-model efforts have begun to jointly pretrain on heterogeneous sources—resting 12-lead ECGs, Holter segments, and wearable signals—to learn representations that transfer across devices and clinical tasks [22]. Additionally, scanned and printed ECGs remain widely used in everyday clinical practice for interpretation and insertion into patient records. 2D ECG records may also be used as source data, provided they are converted to 1D signals and are of high quality comparable to original digital recordings.

### 3.2. Preprocessing of Digital ECG Files: Standardization and Artifact Mitigation

- Purpose and Generalization

Preprocessing of digital ECG files is a foundational step in the machine learning pipeline, with the primary goals of enhancing signal-to-noise ratio (SNR), removing interference, and enabling accurate feature extraction for downstream models. The clinical and computational rationale for preprocessing extends beyond immediate improvements in signal quality: cleaning signals is critical to preventing models from overfitting to noise artifacts, thereby enhancing generalization to new data from external clinical sites. This generalization challenge has become particularly salient in recent foundation-model work, demonstrating that standardized, automated preprocessing is essential to avoid catastrophic performance drops when deploying models to external sites that use different ECG acquisition machines [1,2].

- Band-Pass Filtering for Baseline Wander and High-Frequency Noise Removal

Baseline Wander Removal: Low-frequency components (typically < 0.5 Hz) caused by respiration-induced electrode motion and patient movement are removed using high-pass or band-pass filters. This baseline correction is essential for accurate morphological analysis of the P wave, QRS complex, and T wave, as baseline wander can create false-positive ST-segment deviations [23].

High-Frequency Noise Removal: Powerline interference (50/60 Hz) and muscle activity (EMG) are reduced using low-pass, notch, or adaptive filters. Notch filters specifically target the 50/60 Hz power-line frequency, while low-pass filters with cutoff frequencies typically between 40 and 100 Hz attenuate high-frequency EMG noise without distorting the QRS complex morphology [24].

Combined Filter Strategy: A 0.05–100 Hz band-pass filter is often used as an essential preprocessing step, simultaneously addressing both low-frequency baseline wander and high-frequency noise. This standardized frequency range preserves the diagnostic bandwidth of ECG signals while removing clinically irrelevant artifacts.

- Resampling to Consistent Sampling Frequency

Purpose: ECG signals are often acquired from heterogeneous sources with varying sampling frequencies (e.g., MIT-BIH database sampled at 360 Hz, PTB-XL at 1000 Hz, or commercial systems at 10 kHz). These signals are resampled to a consistent frequency (commonly 200 Hz or 250 Hz) to standardize input for machine learning models [16].

Impact on Computational Efficiency: Resampling reduces computational complexity by decreasing the number of time points per signal, which is important for efficient training of deep learning models, particularly convolutional neural networks and vision transformers that process ECG data as image-like representations. Standardized sampling frequencies also facilitate batch processing and enable the use of fixed-size input tensors.

- Lead Selection and Reordering for 12-Lead Standardization

Standardization Protocol: In 12-lead ECG setups, consistent lead selection or reordering is performed to normalize input for diagnostic models. Specific leads (e.g., Lead II or V1) are frequently selected for arrhythmia detection models, while full 12-lead configurations are retained for comprehensive morphological classification. Reordering ensures that leads are presented in a consistent sequence (e.g., I, II, III, aVR, aVL, aVF, V1-V6) across all datasets, preventing model confusion from variable lead ordering [16].

- Segmentation Strategies: Fixed-Length Windows and Median Beat Analysis

Windowing Approach: Signals are segmented into shorter fixed-length windows (e.g., 10 s) to enable batch processing and reduce memory requirements. This approach is particularly effective for rhythm classification tasks that require temporal context across multiple cardiac cycles [25].

Beat-Level Segmentation: Individual heartbeats are segmented around R-peaks (typically using windows of 200 ms before and 400 ms after the R-peak) for beat-level classification. R-peak detection is performed using algorithms such as Pan–Tompkins or adaptive threshold methods, enabling precise alignment of cardiac cycles for morphological analysis [26].

Median Beat Construction: Using a median filter (e.g., with 200 ms and 600 ms windows) helps estimate and subtract the baseline, producing a clean “median beat” that reflects typical cardiac morphology while reducing beat-to-beat variability and noise. Median beats are particularly valuable for training classification models that require representative cardiac cycle templates [27].

- Alternative Representations: Frequency, Time–Frequency, and Image-Based Transformations

Some preprocessing pipelines transform signals into frequency or time–frequency representations using Fourier transforms or wavelet transforms, capturing spectral features that may be informative for specific arrhythmia detection tasks. Alternatively, signals are transformed into image-like formats via oscillographic rendering for convolutional neural network (CNN) or vision-transformer models, which can leverage pretrained visual architectures for ECG classification. The choice of ECG representation—1D signal, 2D image, or spectral features—depends on the clinical task and the architecture being used, because different encodings emphasize different aspects of morphology and temporal structure, and comparative studies show that performance can vary across representations and downstream objectives [28,29].

- Critical Evaluation and Contemporary Challenges

While the preprocessing pipeline described above represents established best practices, several critical considerations warrant attention. First, the choice of sampling frequency (200 Hz vs. 250 Hz) may introduce information loss when resampling from high-frequency sources (e.g., 10 kHz), potentially affecting the detection of high-frequency QRS components. Second, fixed band-pass filtering (0.05–100 Hz) may not be optimal for all clinical scenarios; for instance, pediatric ECGs or high-resolution ECGs for late potential detection may require wider frequency ranges. Third, R-peak detection algorithms can fail in the presence of significant noise or arrhythmias, introducing segmentation errors that propagate through downstream classification. Finally, the transition from a 1D signal to a 2D image representation may discard temporal precision while introducing artifacts from rendering processes. Recent work emphasizes that preprocessing decisions must be validated across diverse acquisition systems and clinical populations to ensure robust external generalization.

Some pipelines transform signals into frequency or time–frequency representations using Fourier or wavelet transforms, or into image-like formats via oscillographic rendering for CNN or vision-transformer models. Importantly, recent foundation-model work has shown that standardized, automated preprocessing is critical for avoiding catastrophic performance drops when deploying models to external sites that use different ECG machines.

### 3.3. Model Architectures for Deep Learning

Early AI-ECG work relied on classical machine-learning algorithms that operate on handcrafted features derived from the signal, such as intervals, amplitudes, heart-rate variability indices, and wavelet coefficients. Common approaches included logistic regression, support vector machines, random forests, and gradient-boosted trees, which remain attractive in settings with limited data or strong prior domain knowledge because they are relatively sample-efficient and easier to interpret. However, their performance is constrained by the quality and completeness of manually engineered features and their limited capacity to model complex time-dependent relationships.

Supervised and deep learning techniques have largely supplanted traditional feature-engineering methods for high-volume ECG datasets by enabling end-to-end learning from raw or minimally processed waveforms, thereby eliminating manual feature extraction and automatically capturing complex temporal and morphological patterns [30,31,32,33,34,35].

Convolutional neural networks (CNNs) are widely used for arrhythmia classification and the detection of structural heart disease because they can capture local morphological patterns, such as QRS morphology, ST-segment deviations, and T-wave abnormalities, enabling end-to-end learning from raw ECG signals (see Figure 1) without manual feature extraction [16,36,37,38,39,40,41]. Recurrent architectures, including long short-term memory (LSTM) and gated recurrent units (GRU), can model longer-range time relationships and have been applied to rhythm analysis in Holter recordings, though they are gradually being replaced by more scalable architectures such as transformers that enable parallel processing and capture long-range dependencies more efficiently [42,43,44,45,46,47,48]. Hybrid CNN-recurrent models additionally combine local feature extraction with sequence modeling and remain competitive in many tasks [49,50].

**Figure 2:**
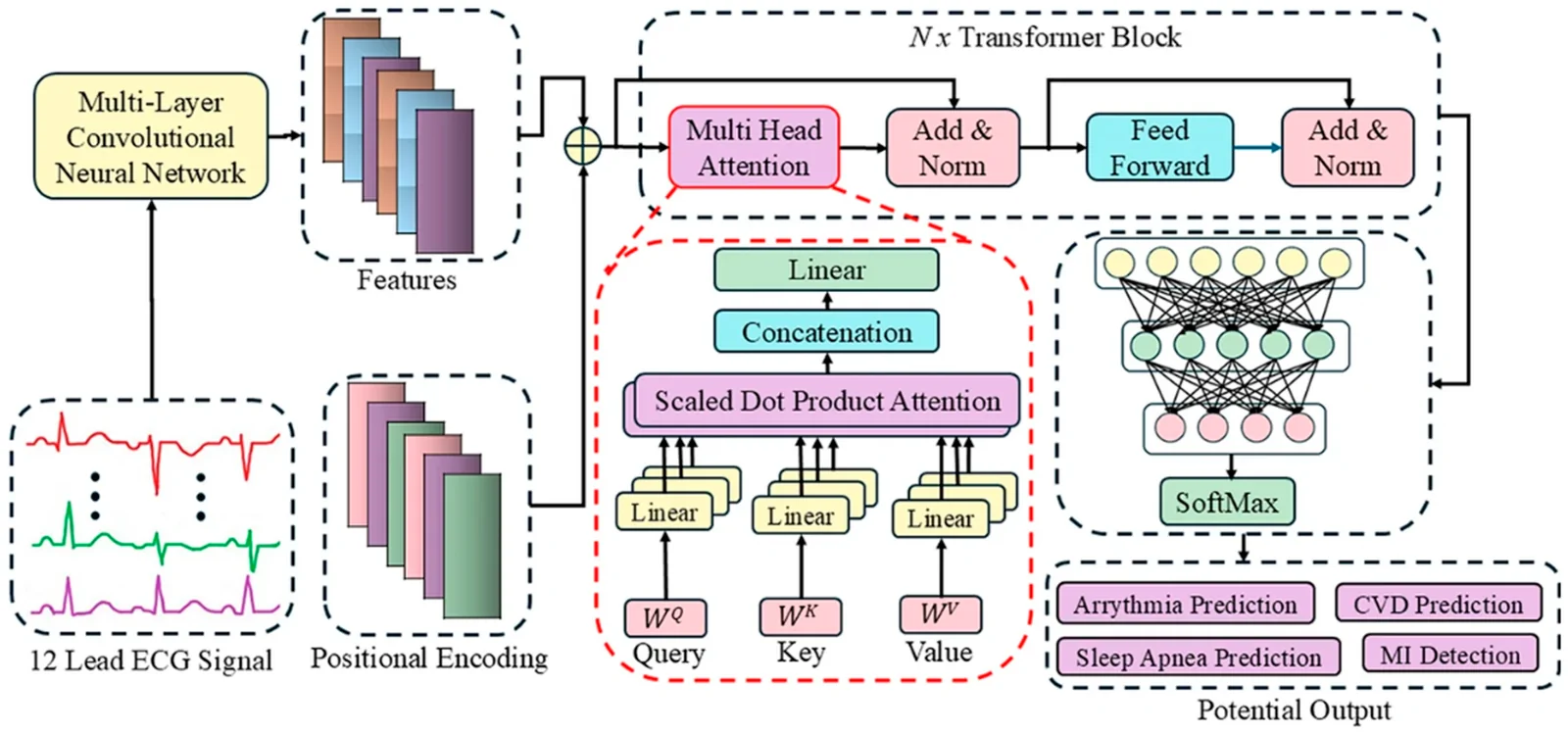
Design example of a transformer-based architecture for ECG analysis and disease prediction.

Transformer-based and foundation-model architectures (see Figure 2) have emerged as the dominant paradigm for AI-enhanced ECG interpretation, leveraging self-supervised learning on large volumes of unlabeled ECG waveforms to create generalizable representations that can outperform or match supervised CNN-based approaches in external validation [51,52]. Self-supervised learning strategies, including contrastive learning, masked-signal modeling, and multi-segment prediction, further reduce reliance on manual labels by pretraining on unlabeled ECG corpora before task-specific fine-tuning [53,54,55,56]. These foundation and self-supervised models exhibit improved label efficiency, cross-dataset generalization, and adaptability compared with task-specific networks [53,54,55,56].

Transformer architectures employ attention mechanisms to capture long-range temporal dependencies across ECG leads, enabling modeling of complex cardiac dynamics spanning multiple heartbeats as shown in Table 1. Recent foundation models such as ECG-FM (90.9 M parameters, Wav2Vec 2.0 architecture), ECG-JEPA (Joint Embedding Predictive Architecture), HuBERT-ECG (Masked Language Modeling), and ST-MEM (Vision Transformer-1D) have demonstrated superior performance on adult ECG interpretation tasks while achieving 3.3–9× higher label efficiency than supervised baselines [57,58]. However, the landscape is heterogeneous: while transformer-based models excel in adult arrhythmia detection, compact structured state-space models like ECG-CPC (3.8 M parameters) dominate pediatric ECG interpretation, cardiac structure and function prediction, and outcome assessment, demonstrating that architectural inductive bias matters more than scale [59]. Knowledge-enhanced models like ECGFM-KED integrate large language models with domain-specific medical knowledge to bridge ECG signal processing with clinical report generation, achieving AUROCs of 0.980–0.992 across 77 cardiac conditions with minimal demographic disparities [60].

Self-supervised learning strategies, including contrastive learning, masked-signal modeling, and multi-segment prediction, further reduce reliance on manual labels by pretraining on unlabeled ECG corpora before task-specific fine-tuning. These foundation and self-supervised models exhibit improved label efficiency, cross-dataset generalization, and adaptability compared with task-specific networks.

### 3.4. AI ECG Model Evaluation

Model evaluation proceeds through a combination of internal and external validation intended to measure performance, generalizability, and fairness. Internally, data are typically split into training, validation, and test sets at the patient level, with cross-validation or temporal splits used to reduce optimistic bias and mimic prospective deployment [16]. External validation in geographically and demographically distinct cohorts is now considered essential, as models often manifest significant performance degradation when applied among different healthcare systems, devices, or prevalence patterns. Recent foundation-model studies have included up to 10–11 external datasets to assess robustness across multiple tasks and institutions [16]. Beyond traditional discrimination metrics such as the area under the receiver-operating characteristic curve, investigators increasingly report calibration, decision curve analysis, and subgroup performance by sex, age, and ethnicity to evaluate clinical utility and algorithmic fairness.

Several studies have performed rigorous multi-site external validation of AI ECG models, revealing substantial but variable performance degradation across institutions. Carter et al. conducted a multicenter external validation of an AI-enabled ECG algorithm (ECG-AI LEF) for detecting low left ventricular ejection fraction across four geographically diverse U.S. health systems (Beth Israel Deaconess Medical Center, Montefiore Medical Center, Monument Health, University of Utah) with 13,960 patients. The algorithm achieved an AUROC of 0.92 (95% CI: 0.91–0.93), sensitivity of 84.5%, and specificity of 83.6%, maintaining performance above 80% across all sites despite using ECG machines from different manufacturers (GE vs. Philips). However, performance varied by age subgroups, with lower odds ratios at the extremes (<40 years: OR 25.8; ≥80 years: OR 16.3 vs. 43.4 in 50–59 years), and inferior performance in patients with a history of heart failure or myocardial infarction. A systematic review of 40 externally validated AI-cardiology studies found mean AUROC decreased by 2.04 percentage points (95% CI: 1.5–2.6%) when comparing internal vs. external validation cohorts. A summary of multi-site validation studies is presented in Table 2. Yao et al.’s EchoNext for structural heart disease detection showed a 5–7 percentage point AUROC drop (from 78–80% internally to external cohorts). For AI-ECG cardiac amyloidosis detection, AUROC remained strong at 0.91–0.93 across external validation cohorts, maintaining performance across age, sex, race, and amyloid subtypes. Richter et al.’s AI-ECG for type 1 heart attack achieved internal test AUC of 0.91 but dropped to 0.85 in external German validation—still outperforming clinicians (0.65) and matching high-sensitivity troponin T (0.87).

**Figure 3:**
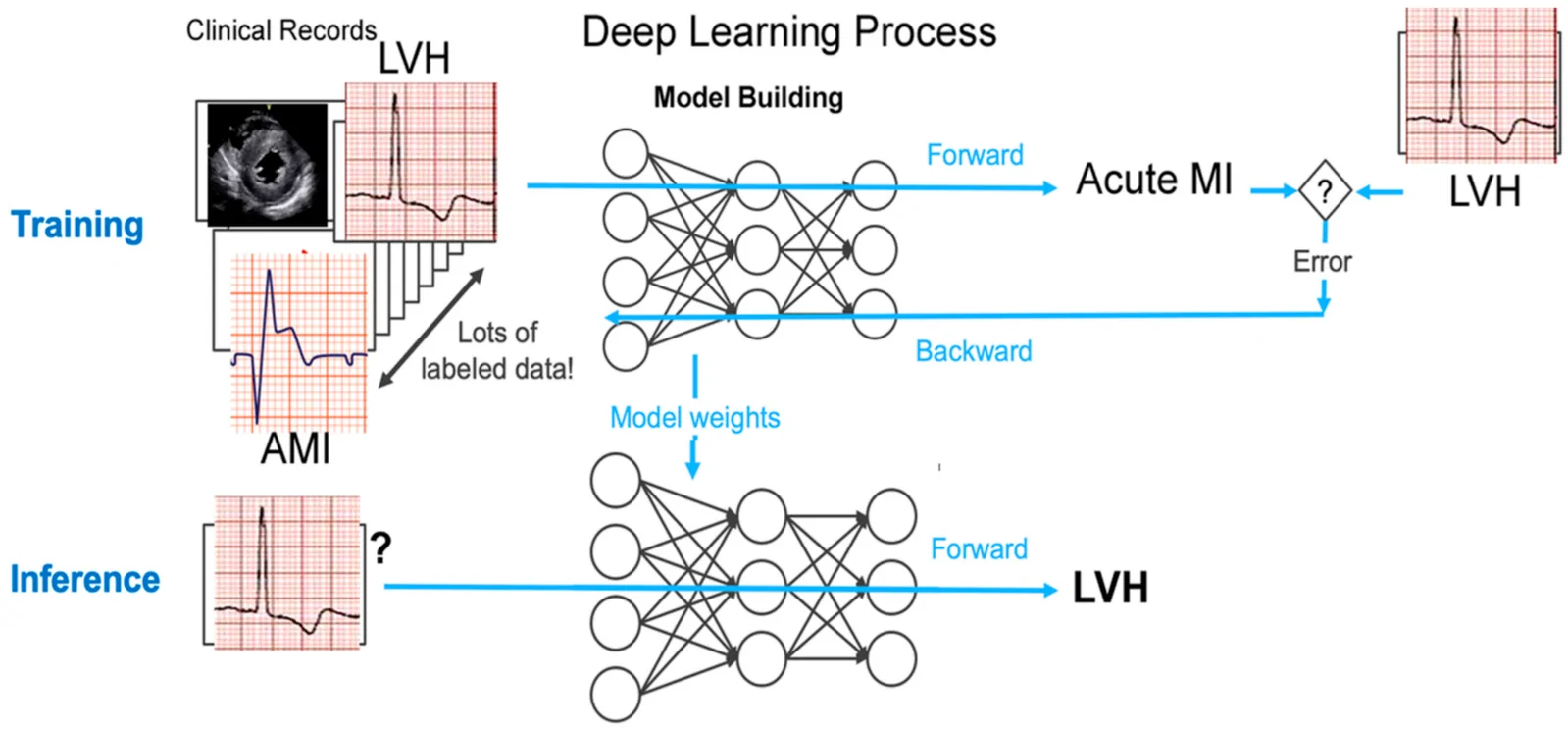
AI ECG Infrastructure. The deep learning process links the features of certain labeled ECGs via forward/backward propagation. The resulting model can now be used for inference. An unknown ECG is forwarded to the model for identification, which results in an LVH finding.

A validated model can be deployed across diverse environments. It is important to recognize that the AI-ECG system workflow typically consists of two components: model training on labeled ECG data and inference on new ECGs for clinical prediction, with users interacting with the trained models [69] as shown in Figure 3.

## 4. Clinical Applications of AI-Enhanced Electrocardiography

Artificial intelligence applied to electrocardiograms has transitioned from experimental research to clinically actionable tools across several areas of cardiovascular medicine (Table 3). By harnessing deep learning techniques, these models extract latent physiological features that are imperceptible to human readers, expanding the ECG’s diagnostic and prognostic potential far beyond traditional rhythm and interval analysis.

Many major academic medical institutions and universities have pioneered AI-ECG clinical research to improve the early detection of heart failure, arrhythmias, and structural heart diseases.

Emerging commercial AI-ECG platforms like Viz.ai, Anumana, Eko Health, and PMcardio are increasingly adopted in academic health centers by leveraging FDA clearances (including De Novo and Breakthrough designations) and integrating via HL7/FHIR protocols to enhance clinical workflows. These platforms offer specialized approaches, ranging from Viz.ai’s passive triage and Anumana’s embedded phenotypic screening to Eko Health’s digital auscultation and PMcardio’s AI-powered digitization of legacy 2D ECGs.

When deploying AI-ECG platforms at scale, academic medical centers prioritize regulatory compliance and seamless Electronic Health Record (EHR) integration, as tools lacking proper clearance cannot support clinical decision-making, and those without deep integration into systems such as Epic or Oracle Cerner disrupt established hospital workflows [1,2,3,4]. Four leading platforms illustrate distinct regulatory milestones and integration frameworks. Viz.ai (Viz Cardio Suite) secured the first FDA De Novo authorization for cardiovascular machine learning notification software (Viz HCM) driving adoption across US academic centers; its workflow operates passively by pulling raw 12-lead ECG data via HL7 feeds from hospital ECG management systems (e.g., GE MUSE, Philips TraceMaster) and sending encrypted high-priority alerts to care teams’ smartphones when critical pathologies such as hypertrophic cardiomyopathy or acute coronary syndrome are detected. Anumana, supported by rigorous Mayo Clinic clinical data, obtained FDA 510(k) clearance for its ECG-AI LEF algorithm for low ejection fraction and the first FDA clearance for a Cardiac Amyloidosis algorithm using standard 12-lead ECGs; it integrates directly into central EHR systems, enabling clinicians to view AI-generated probability scores for heart failure or pulmonary hypertension within the native ECG Viewer widget in Epic during routine chart review. Eko Health’s SENSORA platform holds multiple FDA clearances, including the EFAST algorithm—the first FDA-cleared cardiac foundation model trained on over four million recordings to detect structural heart murmurs and AFib in 5 s—as well as 510(k) clearance for low ejection fraction identification; its bedside integration pairs digital stethoscopes with mobile devices via Bluetooth and syncs digitized ECG strips, heart sounds, and AI risk assessments into Epic or Cerner progression notes through secure FHIR APIs. PMcardio (Powerful Medical, New York, NY, USA) holds full Class IIb certification under EU MDR for widespread clinical use in Europe and received FDA Breakthrough Device Designation for its Queen of Hearts AI model optimized for hidden heart attack detection [83,84] (see Table 4), with active clinical clearance fast-tracked in the US; it addresses legacy infrastructure by enabling clinicians to photograph printed thermal ECG strips with a smartphone app, digitize wave vectors in under 5 s, generate structured PDF diagnostic reports, and export data into EHR databases via secure webhooks.

Diagnostic accuracy metrics for the four emerging AI-ECG platforms, including sensitivity and specificity compared with human cardiologists, are shown in Table 5.

Key Takeaways are summarized in Table 6 above.

AI consistently outperforms cardiologists’ insensitivity, particularly for subtle pathologies (amyloidosis, LEF, paroxysmal AFib).

AI’s specificity advantage is modest but critical for reducing false-positive STEMI activations.

Largest performance gap: Cardiac amyloidosis detection, where AI achieves 86.4% sensitivity versus 47% for cardiologists, reducing diagnostic delay from years to months.

Eko’s EFAST algorithm demonstrates the broadest accuracy across multiple pathologies due to foundation model training on 4+ million recordings.

Human cardiologists remain superior at integrating clinical context, incorporating patient history, symptoms, and comorbidities that AI cannot access.

Viz.ai secured the first FDA De Novo authorization for cardiovascular machine learning notification software, driving adoption across US academic institutions.

Anumana obtained the first and only FDA clearance for a Cardiac Amyloidosis algorithm using standard 12-lead ECGs, a disease traditionally taking years to detect.

PMcardio’s high specificity (95.6%) for ST-MI mimics reduces false-positive STEMI activations, preventing unnecessary catheterization lab deployments.

### Clinical Decision Support

AI-assisted ECG interpretation is increasingly integrated into decision pathways across care settings. In emergency departments, rapid AI-ECG triage tools help clinicians identify acute coronary syndromes, arrhythmias, and heart failure presentations, thereby reducing time to intervention. In outpatient and oncology contexts, AI-ECG models that predict drug-induced QT prolongation or cardiotoxicity may enable earlier therapy modification and individualized monitoring, particularly after QT-prolonging medications or anthracycline-based chemotherapy [72,108,109,110].

Furthermore, AI outputs can prioritize patients for advanced imaging based on latent risk signatures invisible to traditional metrics. Such “ECG-first” triage has been particularly valuable in resource-limited environments. Importantly, clinical adoption emphasizes a collaborative model—AI as an augmentative tool that enhances, rather than replaces, physician judgment. Well-designed interfaces providing explainable features, confidence estimates, and workflow compatibility remain essential for sustained clinical trust [111].

## 5. Emergence of AI ECG Digital Biomarkers

Beyond cardiovascular disorders, AI-ECG has been recognized as a window into systemic physiology [112]. Algorithms have been developed to infer conditions such as obstructive sleep apnea, chronic obstructive pulmonary disease (COPD), and biological aging (“ECG-age”) directly from sinus rhythm recordings [113]. These models use subtle variations in beat morphology, interval variability, and repolarization heterogeneity that reflect autonomic tone and cardiopulmonary load. In predictive cardiology, AI-ECG has been used to estimate the future risk of AF, even in patients with apparently normal rhythms [36]. By identifying subclinical electrical remodeling, these approaches may enable targeted preventive strategies. Additionally, AI-ECG models can estimate biological age and predict future cardiometabolic disease risk, demonstrating utility beyond traditional cardiovascular endpoints. Collectively, they represent the evolution of ECG interpretation from categorical diagnosis to quantitative phenotyping, bridging cardiovascular and systemic health assessment. AI-ECG-generated digital biomarkers demonstrate promising discriminatory performance, but many are still best viewed as screening or triage tools rather than substitutes for definitive diagnosis [95,114,115,116,117].

Table 7 summarizes publicly documented AI-ECG biomarkers spanning cardiovascular, cardiopulmonary, and systemic phenotyping applications. Commercial examples are listed where a vendor-specific program or marketed workflow is publicly documented; otherwise, entries are labeled as research-stage.

## 6. Research Directions and Innovation

Recent progress in artificial intelligence-enhanced electrocardiography is increasingly shaped by four intertwined research areas: scalable pretraining via foundation and self-supervised models, multimodal and integrated architectures, generative and simulation frameworks, and advances in explainability. Together, these developments aim to move beyond narrow, task-specific algorithms toward flexible, trustworthy systems that can be adapted across indications, populations, and healthcare environments.

### 6.1. Modern Foundation Models and Self-Supervised Learning

Foundation models for ECG seek to learn generic, reusable representations from very large corpora of predominantly unlabeled recordings. In contrast to traditional supervised approaches, which require labor-intensive expert annotation for each target task, self-supervised objectives exploit the intrinsic structure of the ECG signal. Typical strategies include masked signal modeling (predicting masked segments from surrounding context), contrastive learning (bringing augmented views of the same beat or lead closer in representation space while pushing different signals apart), and sequence-to-sequence prediction across time scales [118,119,120,121,122]. These objectives encourage the model to encode morphology, rhythm, and temporal dependencies in a way that is not restricted to a single diagnosis.

Once pretrained, the same backbone can be fine-tuned with relatively modest labeled datasets for diverse downstream applications such as arrhythmia classification, detection of structural heart disease, or risk prediction in specific cohorts. This paradigm improves sample efficiency, enabling high performance even with only a few hundred or a few thousand labeled examples available for a new indication. It also supports domain adaptation: by performing a brief fine-tuning step on data from a new institution, vendor, or ethnicity, the model can be recalibrated to local distributions without retraining from scratch. Conceptually, this shifts AI-ECG from single-use algorithms toward a platform model that can be continuously extended to emerging tasks, including rare diseases and niche clinical questions.

### 6.2. Multimodal and Integrated Systems

A second major trajectory involves integrating ECG with complementary data sources within multimodal architectures. The ECG captures high-resolution information about cardiac electrophysiology, but its diagnostic yield and prognostic value can be enhanced when contextualized with clinical covariates. Contemporary models therefore seek to jointly encode 12-lead (or single-lead) waveforms with elements of the electronic health record (EHR), such as demographics, comorbidities, medications, laboratory values, and vital signs, as well as imaging data including echocardiography and cardiac or thoracic computed tomography and magnetic resonance.

In such systems, each modality typically passes through a specialized encoder (e.g., a convolutional or transformer-based time-series encoder for ECG, a text encoder for clinical notes, or a vision backbone for imaging) before being fused in a shared latent space. This design supports multitask learning, in which a single model simultaneously predicts multiple endpoints—for instance, left ventricular function, pulmonary pressures, arrhythmic risk, and short-term clinical deterioration. Multitask setups can exploit shared pathophysiological structure between outputs, often improving calibration and robustness compared with separate single-task networks. Furthermore, cross-modal prediction (e.g., inferring echocardiographic left ventricular ejection fraction or valvular disease directly from ECG plus structured EHR features) enables “virtual imaging triage,” prioritizing patients for resource-intensive tests based on an inexpensive, widely available signal.

### 6.3. Generative and Simulation Models

Generative AI ECG modeling is a new frontier in which synthetic or counterfactual ECGs are used to support interpretation, explanation, data augmentation, and model development. While clinical translation remains in its early stages and requires rigorous validation, it has emerged as a complementary line of research that addresses several limitations of purely discriminative AI-ECG systems. Models such as variational autoencoders, generative adversarial networks, diffusion models, and autoregressive sequence generators are used to synthesize realistic ECG waveforms, either de novo or conditioned on clinical attributes (e.g., rhythm label, QRS duration, presence of hypertrophy) [123,124,125]. Synthetic ECGs can mitigate class imbalance by augmenting underrepresented rhythms or pathologies, and they allow exploration of rare or ethically sensitive scenarios without relying solely on actual patient recordings [126,127].

Beyond simple data augmentation, generative and simulation frameworks support systematic robustness testing. By gradually introducing noise, baseline wander, electrode misplacement, or specific morphological perturbations (e.g., progressive ST-segment elevation or an increasing PR interval), investigators can probe how model predictions change and identify brittle regimes or failure modes. This is particularly valuable for safety-critical tasks, such as detecting life-threatening arrhythmias or ischemia, where adversarial perturbations and distribution shifts may otherwise go unnoticed. In education, curated libraries of synthetic yet physiologically plausible ECGs provide standardized material for clinicians’ training and assessment while avoiding disclosure of identifiable patient data. As generative modeling matures, there is growing interest in using these systems to simulate longitudinal trajectories, enabling in silico trials in which hypothetical interventions or disease progressions are explored before real-world deployment.

### 6.4. Explainability and Interpretability

As AI-ECG models become more complex, explainability has shifted from a largely academic interest to a practical requirement for clinical adoption and regulatory approval. Methods such as saliency maps, Grad-CAM-like approaches adapted to one-dimensional signals, and integrated gradients aim to identify the time points, beats, or leads most influential to a given prediction. Lead-wise relevance scores can help determine whether a model is responding to physiologically plausible features, such as QT prolongation, fragmented QRS complexes, or localized ST-T changes, rather than to artifact or noise. By mapping these patterns onto familiar electrocardiographic concepts, explainability tools provide a bridge between deep learning outputs and established clinical reasoning.

In practice, however, clinician reception has been mixed. Saliency maps and related visualizations are often regarded as post hoc reassurance rather than as tools that materially change clinical decision-making. Usability studies suggest that clinicians find these outputs “somewhat helpful” in improving confidence in AI-based ECG interpretations, but the effect is usually limited to reassurance rather than changes in management. A systematic review of 45 studies found that explainable AI in ECG remains fragmented and insufficiently validated for deployment, with ongoing concerns about stability, computational efficiency, and regulatory readiness. Although feature-level explanations may help clinicians verify AI recommendations, they frequently highlight familiar waveform elements already recognized by human readers, limiting their practical novelty. Likewise, LIME can increase confidence by identifying physiologically relevant ECG segments, but it often functions more as a validation of the expected than as a source of actionable insight. Overall, current explainability methods primarily support trust-building and mitigation of the “black box” problem, but they seldom provide the depth of reasoning needed for true human–machine collaboration in high-stakes cardiology decisions [128,129,130,131].

In parallel, global interpretability approaches aim to explain what a model has learned across an entire dataset rather than focusing on individual predictions. These methods include extracting approximate decision rules from trained networks, fitting simpler surrogate models in the latent space, clustering latent representations into prototype examples corresponding to recognizable ECG phenotypes, and performing counterfactual analyses to assess how predictions change in response to small, targeted input modifications. Importantly, interpretability is increasingly treated as a design constraint rather than an afterthought, with architectures, loss functions, and training curricula being aligned with established electrophysiological principles. When combined with transparent reporting of uncertainty and subgroup-specific performance, these approaches can promote appropriate trust, support shared human–AI decision-making, and facilitate the safe and equitable integration of AI-ECG into routine cardiovascular care [132].

## 7. Practical and Regulatory Challenges

Clinical deployment of AI-enhanced ECG (AI-ECG) confronts substantial practical and regulatory hurdles. These must be addressed to ensure safety, efficacy, and equity across diverse healthcare ecosystems. These challenges coalesce around four interconnected domains: generalization and transferability, algorithmic bias and fairness, data privacy and security, and evolving regulatory frameworks for Software as a Medical Device (SaMD) [133,134].

### 7.1. Generalization and Transferability

Model generalizability is a primary barrier. Algorithms optimized in a specific healthcare environment often exhibit significant performance degradation when deployed elsewhere. This is driven by systematic domain shifts, including heterogeneity in ECG acquisition hardware, filtering protocols, sampling rates, lead-placement conventions, and baseline patient distributions. Mitigation necessitates robust multi-site external validation, adoption of domain-adaptive techniques (e.g., feature alignment, test-time augmentation), and continuous post-deployment monitoring with automated drift detection to maintain performance standards.

### 7.2. Algorithmic Bias and Equity

Algorithmic bias remains a critical concern, with documented performance disparities across sex, age, race, ethnicity, and socioeconomic strata [135]. Training datasets skewed toward majority cohorts often yield reduced sensitivity or specificity for underrepresented populations, reflecting physiologic, anatomic, and pathologic variations. Beyond aggregate metrics such as the area under the receiver operating characteristic curve (AUROC), clinical validation must prioritize subgroup-specific reporting of calibration error, demographic parity, and equalized odds. Strategies including stratified sampling, adversarial debiasing, and mandatory subgroup-specific performance disclosures are prerequisites for equitable deployment thresholds [136].

### 7.3. Privacy, Security, and Data Ownership

ECG signals function as quasi-biometric data, elevating the risk of re-identification from latent representations. Furthermore, adversarial perturbations—subtle, imperceptible waveform alterations—can induce catastrophic misclassifications in deployed systems. Countermeasures must leverage privacy-preserving architectures, including differential privacy during training, homomorphic encryption for inference, and federated learning paradigms (see Table 8) that update models without exchanging raw patient data [137,138]. These frameworks must be balanced against the need for transparent governance regarding intellectual property, patient consent for secondary uses, and clinical liability in breach scenarios.

### 7.4. Regulatory Frameworks and Deployment

Regulatory frameworks for AI-ECG are becoming more defined, but they remain uneven across jurisdictions. In the United States, the FDA’s Software as a Medical Device pathway and, in Europe, the Medical Device Regulation set the baseline for classifying high-risk AI-ECG systems as Class II or III devices. Meeting these requirements increasingly means going beyond retrospective validation to include prospective evidence, preferably randomized where feasible, showing benefit in clinical workflow, patient outcomes, or health economic performance. For adaptive algorithms, safety and effectiveness depend on disciplined lifecycle management: prespecified change protocols, continuous post-deployment surveillance, and human-in-the-loop oversight as models evolve in real-world use. Taken together, these expectations require close collaboration among developers, regulators, and end users to build clinical trust without overstating capability. The FDA’s Predetermined Change Control Plan (PCCP) framework represents a transformative regulatory approach for AI-enabled medical devices, particularly AI ECG systems that require continuous model updates. Released in December 2024 as final guidance titled “Marketing Submission Recommendations for a Predetermined Change Control Plan for Artificial Intelligence-Enabled Device Software Functions,” the PCCP enables manufacturers to implement pre-authorized device modifications without submitting new marketing applications for each change. This framework addresses the fundamental mismatch between traditional medical device regulation and the iterative, self-modifying nature of AI algorithms that evolve through continuous learning [144,145].

The PCCP framework is particularly valuable given the need for continuous improvement based on external validation results, domain adaptation requirements, and performance optimization across diverse populations. Recent work demonstrates that PCCPs can streamline regulatory oversight by reducing the need for new 510(k) submissions while maintaining safety and effectiveness through structured monitoring and validation protocols [146,147,148].

## 8. Integration of AI-ECG into Clinical Practice

Integration of AI-ECG into clinical practice requires interoperability, continuous evaluation, and clinician oversight to ensure that AI augments rather than replaces clinical judgment.

**Figure 4:**
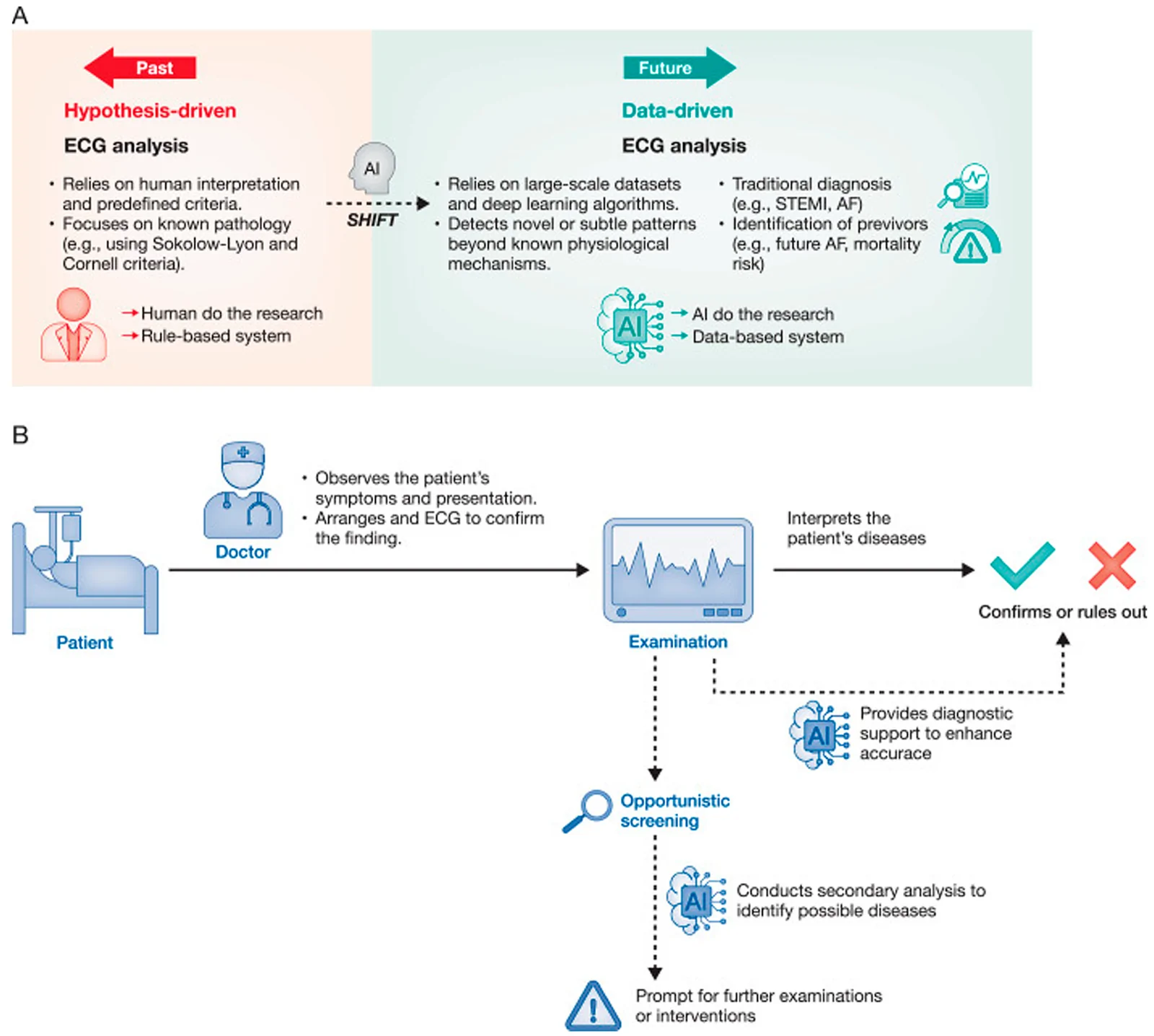
Panel (**A**) illustrates the paradigm shift in ECG analysis enabled by AI. Traditionally, a hypothesis-driven approach is used to establish criteria, and human interpretations are used to identify known pathological markers. In contrast, the data-driven approach harnesses large-scale datasets and deep learning algorithms to detect subtle or novel patterns in ECG signals—those that may not be readily apparent through conventional clinical reasoning. These signatures are used to diagnose conditions such as left ventricular dysfunction and atrial fibrillation and identify “previvors,” individuals with a hidden yet elevated risk for future adverse events. This transformation enables earlier detection of abnormalities, improved risk stratification, and an expanded understanding of cardiac electrical activity beyond historically defined diagnostic criteria. Panel (**B**) illustrates the shift from conventional ECG interpretation to an integrated, AI-driven approach (dashed line), emphasizing how AI serves as a powerful adjunct to traditional methods (solid line) to enhance diagnostic accuracy and patient care [149].

Integration offers improvements in algorithmic performance and new challenges, as it must be tightly integrated into existing workflows, decision structures, and professional roles, as shown in Figure 4 [149].

A central operational paradigm is the use of “dual reporting” models, present AI-generated ECG interpretations alongside physician reports rather than replacing them, allowing automated triage and standardized reads while preserving clinician oversight and contextual adjudication. AI-generated interpretations are presented alongside, but do not replace, physician ECG reports [150,151].

In such configurations, AI systems can pre-classify rhythm, flag potentially critical abnormalities, and generate structured draft text, thereby reducing reporting time for normal or straightforward tracings and helping prioritize complex or high-risk studies. At the same time, the final signed report remains the responsibility of the clinician, who adjudicates discordant findings, contextualizes predictions with clinical information, and can override erroneous or implausible outputs. Early experience suggests that dual reporting can improve throughput and consistency in high-volume settings, but may transiently increase workload during the adoption phase if interfaces are poorly integrated with existing ECG management systems or if alert thresholds are not appropriately calibrated.

The successful implementation of AI-ECG systems requires physicians’ acceptance and a comprehensive understanding of the complete architecture through which signals are acquired, processed, interpreted, and ultimately delivered as clinical reports. Table 9 summarizes the integration capabilities of emerging commercial AI ECG systems.

These AI ECG products typically integrate with EMR/EHR workflows in one of three ways: direct delivery into the chart, API/FHIR-based interoperability, or report export/import through middleware or platform portals.

## 9. Limitations of AI ECG

Despite strong diagnostic performance, the use of AI-ECG in clinical practice is constrained by dataset shift, subgroup bias, limited interpretability, regulatory barriers, workflow integration challenges, and the need for prospective validation of patient-centered benefits. A major concern is overfitting, particularly when models are trained on homogeneous cohorts or optimized on small datasets, which can inflate apparent performance while reducing robustness in external populations [152]. Related to this, external validation failures remain common; performance often declines when models are tested across different hospitals, devices, or patient distributions, with clinically meaningful shifts in sensitivity and specificity after external testing [10,153]. Another important issue is label noise, because ECG ground truth is frequently derived from imperfect clinical documentation, automated statements, delayed adjudication, or surrogate labels rather than uniform expert-confirmed endpoints, thereby introducing misclassification into both training and evaluation sets. Reproducibility is also limited, as many studies do not release code, preprocessing pipelines, or sufficiently detailed calibration thresholds, making it difficult to replicate results or compare models head-to-head across institutions [153,154]. In parallel, regulatory uncertainty persists because AI-ECG tools may not fit neatly into existing device pathways, and questions remain regarding software updates, post-market surveillance, liability, and the evidentiary standard required for clearance or adoption in routine care. Finally, implementation barriers continue to slow real-world use, including variability in ECG formats, poor interoperability with electronic health records, the need for clinician training, workflow disruption, cybersecurity concerns, and challenges in integrating outputs into point-of-care decision-making. As a result, the field now requires more rigorous multicenter external validation, standardized reporting, transparent model sharing, and implementation studies that assess not only accuracy but also usability, safety, and equity in everyday clinical practice [10,85,152,153,154,155].

## 10. Future Directions and Research Priorities for AI ECG

The future of AI-enhanced electrocardiography hinges on three interconnected priorities, algorithm generalizability, implementation science, and clinical utility, with agentic AI emerging as a transformative paradigm. First, future AI models must prioritize algorithmic generalizability by training on diverse, multicenter datasets that encompass varied demographics, ECG machines, and clinical settings to ensure robustness across populations. Second, successful implementation requires seamless workflow integration, drawing on implementation science and user-centered design principles, to ensure AI outputs are delivered at the point of care without disrupting existing clinical routines. Third, pragmatic trials must demonstrate the utility of the algorithm—showing that AI actually improves patient outcomes, reduces costs, or enhances efficiency rather than simply achieving high accuracy metrics.

Agentic AI is an emerging technology that goes beyond passive prediction to enable autonomous, goal-directed clinical workflows. Unlike traditional AI that generates predictions for human review, agentic AI systems actively monitor patient status, synthesize heterogeneous data sources (EHRs, lab values, ECG, wearable sensors), and take constructive actions toward defined clinical goals [156]. Recent developments include MARCUS, an agentic multimodal vision-language model designed for end-to-end ECG interpretation, and agentic frameworks that can autonomously analyze complex medical data, personalize treatment plans, and support real-time decision-making. By 2026, predictions suggest that more than 30% of clinical decisions in developed countries will include agentic AI.

Critical emerging priorities include multimodal AI approaches that integrate ECG data with echocardiography, laboratory values, and natural language processing of electronic health records to achieve diagnostic performance beyond that of any single modality. Agentic AI particularly excels here by combining predictive analytics with real-time patient feedback to propose individualized care plans and dynamically adjust treatment based on monitoring data. Explainable AI must evolve from post hoc reassurance to genuine clinical reasoning that helps clinicians understand and trust AI predictions, particularly for detecting subtle pathologies like cardiac amyloidosis or predicting sudden cardiac death. Finally, federated learning and edge computing will enable privacy-preserving model training across institutions while reducing computational costs, addressing both data security and scalability challenges. The Heart Rhythm Society’s 2026 scientific statement provides a comprehensive framework for responsible AI adoption, including agentic systems, emphasizing lifecycle evaluation, governance structures, and ethical principles, including transparency, bias mitigation, and patient privacy as essential for sustainable deployment [157,158,159,160,161].

## 11. Conclusions

AI-ECG has rapidly evolved from a technical proof of concept to a clinically promising tool for cardiovascular diagnostics, particularly in arrhythmia detection and screening for structural heart disease. In addition to identifying rhythm disorders and occult cardiomyopathies, AI-ECG is increasingly being explored as a broader digital biomarker for risk prediction and the detection of systemic disease. The AI ECG’s major strength lies in its ability to extract clinically relevant information from a widely available signal that is underutilized in conventional interpretation. These advances reflect the growing potential of machine learning to augment electrocardiography beyond human visual inspection and rule-based automation.

At the same time, current evidence supports a cautious and balanced view. Many reported models perform well on development datasets, but clinical translation requires stronger external validation, calibration, assessment of subgroup performance, and evaluation across diverse settings and populations. Concerns regarding overfitting, algorithmic bias, label noise, and limited reproducibility remain important barriers to generalizability. In parallel, regulatory uncertainty, data governance challenges, and workflow integration issues continue to complicate implementation in routine care. For AI-ECG to achieve meaningful clinical impact, future studies must move beyond discrimination metrics alone and demonstrate prospective benefit, usability, safety, and equity in real-world practice.

Future clinical implementations should be guided by external validation, subgroup performance assessment, calibration, explainability, clinical workflow testing, and regulatory oversight.

Overall, AI-ECG is best viewed not as a replacement for clinician interpretation, but as a decision-support technology with substantial potential to improve efficiency, consistency, and access to cardiovascular care. With rigorous validation, transparent reporting, and thoughtful integration into clinical workflows, AI-ECG may become an important component of future precision cardiovascular medicine.

## Figures and Tables

**Table 1:** Transformer models used in ECG Analysis.

Model	Architecture	Architecture	Pretraining Method	Training Data Size	Parameters	Adult ECG Interpretation (AUROC)	Adult ECG Interpretation (AUROC)	Pediatric ECG (AUROC)	Pediatric ECG (AUROC)	Label Efficiency (× Improvement)	Label Efficiency (× Improvement)	Label Efficiency (× Improvement)	Key Strengths	Key Limitations
**ECG-FM**	**ECG-FM**	Transformer (Wav2Vec 2.0)	MLM + sequence-level contrastive loss	1.5 M ECGs	90.9 M	90.9 M	0.92	0.92	0.83	0.83	4.2×	Large-scale representation, multi-task capability	Large-scale representation, multi-task capability	Requires substantial computational resources
**ECG-JEPA**	**ECG-JEPA**	Transformer (JEPA)	Joint Embedding Predictive Architecture	174 k samples	~10 M	~10 M	0.91	0.91	0.81	0.81	9.0×	Most label-efficient, frozen feature extraction	Most label-efficient, frozen feature extraction	Limited pediatric performance
**HuBERT-ECG**	**HuBERT-ECG**	Transformer	Masked Language Modeling (MLM)	9.1 M samples	~50 M	~50 M	0.90	0.90	0.80	0.80	5.1×	Large aggregated dataset, strong generalization	Large aggregated dataset, strong generalization	Slower training convergence
**ST-MEM**	**ST-MEM**	ViT-1D Transformer	Masked Autoencoder (MAE)	174 k samples	~12 M	~12 M	0.89	0.89	0.79	0.79	3.8×	Vision transformer architecture, linear evaluation robust	Vision transformer architecture, linear evaluation robust	Poor frozen evaluation performance
**ECG-CPC**	**ECG-CPC**	Structured State-Space Model (SSM)	Contrastive Predictive Coding (CPC)	10.7 M samples	3.8 M	3.8 M	0.88	0.88	0.92	0.92	6.2×	Dominates pediatric & cardiac structure tasks, compact, resource-efficient	Dominates pediatric & cardiac structure tasks, compact, resource-efficient	Underperforms with linear head; requires non-linear pooling
**ECGFounder**	**ECGFounder**	CNN (RegNet-inspired)	Supervised pretraining	10.7 M samples	~25 M	~25 M	0.93	0.93	0.82	0.82	3.3×	Best adult ECG performance, strong frozen extraction	Best adult ECG performance, strong frozen extraction	CNN limitations for long-range dependencies
**MERL**	**MERL**	ResNet18 + Transformer	Weak supervised + contrastive	800 k samples	~20 M	~20 M	0.87	0.87	0.78	0.78	4.5×	Multi-task contrastive learning	Multi-task contrastive learning	Inferior to transformer models
**ECGFM-KED**	**ECGFM-KED**	ResNet + LLM	Weak supervised + contrastive (signal-text)	800 k samples	~30 M	~30 M	0.94	0.94	0.84	0.84	5.8×	Knowledge-enhanced, clinical report generation (AUROC 0.980–0.992, 77 conditions)	Knowledge-enhanced, clinical report generation (AUROC 0.980–0.992, 77 conditions)	Requires LLM integration, complex deployment
**Net-1D** (baseline)	**Net-1D** (baseline)	CNN (1D)	Supervervised (scratch)	Variable	~15 M	~15 M	0.85	0.85	0.76	0.76	1.0×	Simple, fast training	Simple, fast training	Poor generalization, inferior to FMs
**S4** (baseline)	**S4** (baseline)	Structured State-Space	Supervervised (scratch)	Variable	~10 M	~10 M	0.86	0.86	0.77	0.77	1.0×	Captures long-range dependencies	Captures long-range dependencies	Lower label efficiency than FMs

**Note:** AUROC values represent average performance across 26 clinically relevant tasks from 12 public datasets (1650 regression/classification targets). Adapter metrics are from fine-tuning evaluation; ECG-CPC and ECG-JEPA maintain strong frozen performance. Label efficiency is measured as the training sample reduction to match the S4 baseline performance [61,62,63].

**Table 2:** Summary of Multi-Site Validation Studies.

Study	Model/Algorithm	Task	Development AUROC	External AUROC	Performance Drop	Validation Sites	Sample Size
Carter et al. 2026 [64]	ECG-AI LEF	Low ejection fraction	0.92	0.92	0% (stable)	4 US health systems	13,960
Chen et al. 2026 [65]	Systematic review (40 studies)	Multiple	varies	varies	2.04% avg	40 studies	var
Poterucha et al. 2025 [66]	EchoNext	Structural heart disease	0.78–0.80	0.71–0.73	5–7%	NYP multicenter	12,847
Anumana et al. 2024 [67]	AI-ECG CA	Cardiac amyloidosis	0.93	0.91	2%	3 US centers	10,127
Büscher et al. 2025 [68]	AI-ECG ACS	Type 1 heart attack	0.91	0.85	6%	2 US + 1 German	8500
Attia et al. 2019 [69]	AI-ECG LVSD	LV systolic dysfunction	0.93	0.89	4%	4 US centers	44,959

**Table 3:** AI-ECG Research at Leading Academic Institutions.

Mayo Clinic	Anumana (Commercial Spinoff)	Low Ejection Fraction, Atrial Fibrillation Detection	Foundational AI Algorithms for Hidden Cardiac Conditions; Commercialized Through Anumana	[70,71,72]
Columbia University & NewYork-Presbyterian	EchoNext	Structural heart disease screening	AI-powered screening tool integrated into workflows; analyzes ordinary ECG data to identify high-risk patients	[73]
Cleveland Clinic	Real-time AI-ECG algorithms	Hypertrophic Cardiomyopathy (HCM) flagging	Real-time AI flags patients for potential HCM; enables faster follow-ups and diagnosis in undifferentiated populations	[74]
Yale University (CarDS Lab)	ECG Dx	Future heart failure risk, rhythm/conduction disorders	AI-ECG diagnostic models identifying future heart failure risks; complex rhythm and conduction disorder diagnoses	[75,76]
UCSF	Explainable AI algorithms	12-lead ECG diagnostic analysis	Explainable AI achieving diagnostic accuracies comparable to expert cardiologists	[77,78]
Rutgers University	AI-based ECG algorithms	Early heart disease detection	Multi-year collaborative initiative; low-cost early detection of heart diseases	[79]
Imperial College London (NHLI)	AI-ECG model suite	Heart failure, valve disease detection	Suite of AI-ECG models deployed in hospitals for heart failure and valve disease	[80]
Mount Sinai	Deep learning ECG models	Expanded diagnostic capabilities	Deep learning models enabling AI to read ECGs like language models process text	[81,82]

**Table 4:** Summary of regulatory status and EHR integration.

Viz.ai (Viz Cardio)	Viz.ai (Viz Cardio)	Cleared (De Novo for Viz HCM)	Cleared (De Novo for Viz HCM)	Certified (MDR compliant)	Certified (MDR compliant)	Epic & Cerner Native Integration via FHIR APIs; mobile app push notifications [4,5,6,7,8,9,10,15,16,36,85]
Anumana (inference/Mayo)	Anumana (inference/Mayo)	Cleared (510k for Low EF; Breakthrough for Amyloidosis)	Cleared (510k for Low EF; Breakthrough for Amyloidosis)	In Progress/Select certifications	In Progress/Select certifications	Web-based ECG Viewer and direct Epic integration via HL7/FHIR [4,5,6,7,8,9,10,15,16,36,85]
Eko Health (SENSORA)	Eko Health (SENSORA)	Cleared (510k for EFAST, Low EF, AFib)	Cleared (510k for EFAST, Low EF, AFib)	Certified (Class IIa/IIb)	Certified (Class IIa/IIb)	Mobile App & EHR Sync; pushes point-of-care auscultation data into patient chart [4,5,6,7,8,9,10,15,16,36,85]
PMcardio (Powerful Medical)	PMcardio (Powerful Medical)	Breakthrough Designation (Pending full 510k)	Breakthrough Designation (Pending full 510k)	Certified (Class IIb under EU MDR)	Certified (Class IIb under EU MDR)	Standalone Mobile Interface/API; smartphone camera scans bridge physical paperwork to digital records [4,5,6,7,8,9,10,15,16,36,85]

**Table 5:** Diagnostic Accuracy Metrics: AI-ECG Platforms vs. Human Cardiologists.

Platform	Pathology	AI Sensitivity	AI Specificity	Cardiologist Sensitivity	Cardiologist Specificity	AI Improvement (Sens)	AI Improvement (Spec)
Viz.ai [86,87,88]	Hypertrophic Cardiomyopathy (HCM)	89.5%	92.3%	72.1%	85.4%	+17.4%	+6.9%
Viz.ai [89,90]	Acute Coronary Syndrome (ACS)	91.2%	88.7%	83.5%	86.1%	+7.7%	+2.6%
Anumana [91,92,93,94]	Low Ejection Fraction (<45%)	83.0%	85.0%	65.0%	78.0%	+18.0%	+7.0%
Anumana [95]	Cardiac Amyloidosis	86.4%	91.2%	47.0%	82.0%	+39.4%	+9.2%
Eko Health [96,97,98,99,100,101,102,103]	Structural Heart Murmurs	93.5%	91.8%	78.2%	87.3%	+15.3%	+4.5%
Eko Health	Atrial Fibrillation (AFib)	96.7%	94.2%	89.1%	91.5%	+7.6%	+2.7%
Eko Health	Low Ejection Fraction	81.5%	84.3%	63.8%	76.9%	+17.7%	+7.4%
PMcardio [84,104]	STEMI/Heart Attack	94.8%	92.1%	86.3%	89.4%	+8.5%	+2.7%
PMcardio [105,106,107]	ST-Elevation MI Mimics	88.2%	95.6%	79.5%	87.8%	+8.7%	+7.8%

**Table 6:** Table Summary Averages.

Overall Sensitivity	89.0%	72.0%	+17.0%
Overall Specificity	91.0%	85.0%	+6.0%
Best Sensitivity Gain	Cardiac Amyloidosis	—	+39.4%
Best Specificity Gain	ST-MI Mimics	—	+7.8%

**Table 7:** Overview of AI-ECG biomarkers.

Reduced left ventricular ejection fraction (LVEF less than or equal to 40%)	Left ventricular systolic dysfunction/heart failure risk	Anumana ECG-AI LEF	FDA-cleared/commercial	Screening, triage, referral	Public indications for use specify detection aid from a standard 12-lead ECG and note it is not a standalone diagnostic device.
Low ejection fraction/low LVEF	Left ventricular systolic dysfunction	Mayo Clinic/Anumana implementation studies	Clinical implementation research	Screening and workflow integration	Multicenter implementation studies show translation of AI-ECG CDS into routine care pathways.
Pulmonary hypertension	Elevated pulmonary vascular load	Anumana ECG-AI PH	Commercial/publicly documented	Screening and triage	Listed by the company among currently available ECG-AI algorithms.
Cardiac amyloidosis	Infiltrative cardiomyopathy	Anumana ECG-AI CA	FDA-cleared/commercial	Screening and triage	Company reports FDA clearance and availability for clinical use from a standard 12-lead ECG.
Cardiac amyloidosis	Infiltrative cardiomyopathy	AccurKardia	Emerging commercial program	Screening	Publicly documented as an AI-driven ECG detection program with patent coverage.
Atrial fibrillation from sinus rhythm	Future or occult AF risk despite sinus rhythm	Research-stage; integrated into broader ECG analytics ecosystems	Research/translational	Screening and preventive risk stratification	Landmark AI-ECG work showed AF detection from sinus-rhythm ECGs, supporting subclinical electrical remodeling as a biomarker.
AF burden/arrhythmia event classification	Ambulatory rhythm burden and event adjudication	Philips Cardiologs	Commercial	Ambulatory monitoring and workflow support	Philips documents AI-assisted ECG and ambulatory rhythm analysis for clinical workflows.
Rhythm-event triage/false-positive reduction	Ambulatory ECG monitoring efficiency	Philips Cardiologs	Commercial	Monitoring workflow optimization	Commercial focus is rapid and scalable AI-assisted rhythm interpretation rather than a single disease-specific biomarker.
Structural heart disease	Broad structural cardiac abnormality phenotyping	Research-stage AI-ECG models	Research	Screening and phenotyping	Reviews describe AI-enhanced ECG for detection of structural heart disease and disease management.
Hypertrophic cardiomyopathy	Myocardial structural disease	Research-stage AI-ECG models	Research	Screening and case finding	Multicenter validation studies have shown AI-ECG detection of hypertrophic cardiomyopathy.
Aortic stenosis	Valvular outflow obstruction	HeartSciences/AccurKardia programs	Emerging commercial/pre-clearance public programs	Screening	Public sources document breakthrough-device or patent activity around ECG-based aortic stenosis and related valvular detection efforts.
Hyperkalemia	Electrolyte abnormality	AccurKardia AK+ Guard and related programs	Emerging commercial	Screening and triage	Public company materials document ECG-based electrolyte detection programs within commercial pipelines.
Obstructive sleep apnea	Sleep-disordered breathing phenotype	Research-stage AI-ECG models	Research	Screening	Reviews of AI-enhanced ECG note nontraditional targets including systemic and cardiopulmonary conditions beyond classical rhythm diagnosis.
COPD/chronic lung disease phenotype	Cardiopulmonary burden	Research-stage AI-ECG models	Research	Screening/phenotyping	Reviews describe AI-ECG as a window into cardiopulmonary physiology beyond classical cardiovascular endpoints.
ECG-age/biological age	Physiologic or electrical aging	Research-stage AI-ECG models	Research	Risk stratification	AI-ECG reviews describe biological age estimation and latent physiologic phenotyping from ECGs.
Cardiometabolic risk	Future cardiometabolic disease risk	Research-stage AI-ECG models	Research	Prevention/triage	Reviews describe prediction of broader disease risk and systemic phenotypes using AI-enhanced ECG.
Mortality/global cardiovascular vulnerability	Composite outcome or latent physiologic risk	Research-stage and platform models	Research	Prognostic enrichment	AI-ECG reviews report outcome prediction applications extending beyond categorical diagnosis.
Digital electrophysiologic phenotype panels	High-dimensional ECG biomarker signatures	ECGomics platform	Research platform	Discovery, association mapping, biomarker research	ECGomics is presented as an open platform for AI-ECG digital biomarker discovery and benchmarking.

**Table 8:** Mitigation Strategy Comparison.

Mitigation Strategy	AUROC Improvement	Key Study	Mechanism
Domain Adaptation	3–8%	Liu et al. 2023 [139]	Fine-tunes model on target domain data
Federated Learning (FedAvg)	Maintains 95–98% centralized	Phan et al. 2024 [140]	Distributed training without data sharing
Federated Domain Adaptation (FedGP)	6–12% target domain	Wang et al. 2024 [141]	Gradient projection with auto-weighting
Test-Time Augmentation (TTA)	1–3%	Sun et al. 2024 [142]	Inference-time augmentation averaging
Source-Free Domain Adaptation (SFDA)	4–6%	Zhang et al. 2024 [143]	Pseudo-labeling without source data

**Table 9:** Commercial AI ECG Systems connectivity.

System	Typical EMR/EHR Workflow Integration	Evidence from Sources
AliveCor Kardia 12L	Used as a clinical ECG acquisition/interpretation tool; results are intended to fit into clinician workflows, with FDA-cleared cardiac determinations and commercial deployment in care settings.	Product pages emphasize clinical use and workflow in primary care and acute settings.
Philips Cardiologs	Integrates as an ECG analysis platform for resting/ambulatory ECG interpretation, with clinical output that can be incorporated into cardiology workflows and record systems.	Philips describes AI-assisted ECG analysis and FDA/CE-cleared status.
iRhythm Zio/ZEUS	Sends analyzed ECG findings from wearable monitoring into clinical review workflows; the AI is positioned to speed comprehension of large ECG datasets.	iRhythm markets FDA-cleared AI and CE-marked Zio/ZEUS ecosystem for large-scale ECG analysis.
Eko SENSORA/EFAST	Works as a point-of-care diagnostic workflow where simultaneous ECG and heart sounds are analyzed and results support clinician assessment during the encounter [16,17]	Eko describes FDA-cleared software that analyzes ECGs and heart sounds in real time [16,18]
Powerful Medical PMcardio	Commonly deployed through clinical workflows as AI ECG interpretation software; integration details are product- and deployment-specific, with CE-certified clinical use in Europe.	PMcardio advertises instant AI ECG interpretations and clinical validation.
Cardiomatics	Cloud-based ECG analysis that typically fits into ambulatory/Holter review workflows, where interpreted reports can be shared back to the clinician and chart.	Cardiomatics is described as CE/MDR-certified software for analyzing ambulatory ECG tests.

## Data Availability

No new data were created or analyzed in this study. Data sharing is not applicable to this article.
